# Change of Location

**Published:** 1916-03

**Authors:** 


### Change of Location.

Dr. W. H. Summit and family have moved to North Fort Worth from Strawn.

Dr. T. R. Beech, of Atlanta, Ga., has located in Glazier for the purpose of practicing medicine.

Dr. D. B. Beach has moved to Dodsonville.

Dr. L. T. Smith, of Port Bolivar, has moved to Rio Vista, where he has formed a partnership with Dr. J. M. Meason.

Dr. L. S. Chenoweth, of Fort Stockton, has moved his office from the Stockton building to the building formerly occupied by the First National Bank.

Dr. Henry Bradbrook, of Wardsworth, has moved to Brookshire to engage in the practice of his profession.

Dr. Silas Ballard, formerly of Temple, late of El Paso, is located at Vernon. The doctor is an eye, ear, nose and throat specialist.

Dr. William D. Ray, of Houston, has filed license to practice medicine with District Clerk Rudd, of Marshall.

Dr. W. A. Bishop, accompanied his brother to Dallas recently to put him in a sanitarium.

Dr. J. W. Harrison, of Houston, has moved to Palacios to engage in the practice of his profession.

Dr. J. H. Davis, of Colorado City, has moved to Abilene.

Dr. Lewis Mackey has moved to Cooper and has opened office rooms for the present at the Ross Hotel.

